# Alternative mating tactics in brown widow spiders: mating with or without male self-sacrifice does not affect the copulatory mechanism

**DOI:** 10.1186/s12983-025-00560-8

**Published:** 2025-04-02

**Authors:** Lenka Sentenská, Dante Poy, Maydianne C. B. Andrade, Gabriele B. Uhl

**Affiliations:** 1 Zoological Institute and Museum; General and Systematic Zoology, University of Greifswald, Loitzer Strasse 26, 17489 Greifswald, Germany; 2 Department of Biological Sciences, University of Toronto Scarborough, 1265 Military Trail, Toronto, ON M1C 1A4 Canada; 3 Department of Botany and Zoology, Masaryk University, Kotlářská 2, Brno, 611 37 Czechia; 4 Division of Arachnology, Museo Argentino de Ciencias Naturales—CONICET, Buenos Aires, Argentina

**Keywords:** Araneae, Copulation, Immature mating, Microtomography, Cannibalism

## Abstract

**Background:**

Male self-sacrifice during mating is one of the most extreme forms of male reproductive investment. In two species of widow spiders (genus *Latrodectus*), males trigger sexual cannibalism by “somersaulting” into the fangs of the female after copulatory coupling is achieved. In this position, sperm are transferred with the secondary sexual organs, the transformed pedipalps of the male, while the female starts feeding on his opisthosoma. In *Latrodectus hasselti* and *L. geometricus*, matings also occur with subadult females (i.e. females in their last moulting stage) but during these “immature” matings, males do not perform the somersault. Consequently, mating positions differ dramatically between matings with adult and subadult females. Here, we investigate the copulatory mechanism of adult and immature matings in the brown widow *L. geometricus* by shock-freezing copulating pairs and 3D X-ray microtomography*.* We hypothesize differences in the copulatory mechanism and depth of insertion of the sperm transfer structures between the two mating tactics.

**Results:**

We found that the copulatory mechanism does not differ between adult and immature mating tactics and do not depend on whether a somersault occurs. Furthermore, the somersault does not improve intromission depth of the male sperm transfer organs into the female sperm storage organs.

**Conclusions:**

Our results suggest that the somersault has evolved solely due to the selective advantages of sexual cannibalism. The costs and benefits of both mating tactics need to be further explored using paternity studies in order to understand their relative adaptive value.

## Background

It is well documented that genitalia and related secondary sexual structures can be highly diverse and complex, particularly among species with internal fertilization [1, 2]. Coevolution of male and female genitalia has been shown to be influenced by ecological factors (e.g., predation risk), evolutionary divergence between related taxa (e.g., lock-and-key barriers to hybridization), sexual conflict, and sexual selection [1–7]. Much less understood is how genital morphology and mating behaviour interact, even though the interaction is likely to be a critical component of successful mating and fertilization [8]. For example, male guppies court using a conspicuous sigmoid swimming pattern that attracts receptive females facilitating genital coupling. However, males also approach unreceptive females without courtship, and attempt an unsolicited copulation using a rapid thrust of their intromittent organ (gonopodium), which can lead to selection for longer gonopodia [9]. The alternative mating tactic of male guppies has been used across a number of studies to explore trade-offs among genital coupling, sexual selection and conflict, and their evolution under varied ecological conditions (see [10]). This is one well known exception to a dearth of studies that consider the interdependence of genital morphology and behaviour [8, 11]. Studying the diverse biomechanics of genital coupling, combined with the transient nature of copulatory behaviours can be challenging [8, 11]. However, understanding the co-evolution of genital organs and mating behaviour necessarily requires understanding the biomechanic aspects of mating [2]. Here, we study the mechanics of genital coupling and whether it is affected by alternative male mating behaviours in brown widow spiders (*Latrodectus geometricus*), using computed microtomography to visualize male and female genitalia when in copula.

Studying links between genital mechanics and behaviour in spiders is particularly interesting for a number of reasons. First, there is wide variation in genital structures and conformational changes during copulation, and genitalic characters are often used for taxonomy, suggesting that species-specific lock-and-key structures may be common [4, 12]. Second, variation in copulatory behaviours and postures is notable across taxa, with different families varying in the orientation of male and female during mating [13, 14]. Third, spider males possess two copulatory organs located on the terminal part of their leg-like appendages, the pedipalps, and females possess paired sperm storage organs (i.e. spermathecae). Thus, to transfer sperm to both spermathecae (and so decrease the risk of sperm competition), the male must copulate twice, once with each copulatory organ [1, 12, 15]. Fourth, males of many spider species leave mating plugs or sperm plugs inside female genitalia (some of which are formed from broken portions of the male’s genitalia). The placement of plugs can determine their efficacy and can be affected by copulatory mechanics and behaviour [16]. Fifth, the possibility of sexual cannibalism has given rise to a range of male copulatory behaviours that reduce the risk of female attacks but may also impose selection on genital structures and copulation itself (e.g., extremely rapid sperm transfer of some species, [17, 18]).

Within the genus *Latrodectus* (Theridiidae) genital structures are coiled in males and females, with complementary forms [19]. Moreover, there are two species of *Latrodectus* in which complicity in sexual cannibalism is part of the male mating system and cannibalism during copulation appears to be triggered by male behavior. In both *Latrodectus geometricus* and *L. hasselti*, when the genitalia are coupled, the male raises himself to a headstand position and then flips his body over bringing its dorsal side in contact with the mouthparts of the female [20, 21]. Female cannibalism of the male begins while the male is in this ‘copulatory somersault’ position and continues while sperm is transferred [20]. Thus, although sexual cannibalism is typically perceived as a behaviour that is highly disadvantageous for males [22], it is considered to be a general component of the mating system in these species and others with similar extreme patterns of reproductive investment [23–25]. In *L. hasselti*, it has been argued that the copulatory somersault has evolved due to benefits in terms of paternity and survivorship of offspring of cannibalised males [26, 27]. For *L. geometricus* it is unclear whether cannibalism is advantageous for the males [28]. However, in both species the alternative hypothesis that the somersault itself is beneficial, and cannibalism is simply a correlated outcome, has not yet been tested.

There are several reasons to hypothesize that the copulatory somersault behavior might affect fertilization success via changes in the biomechanics of genital coupling. The portion of the *Latrodectus* male’s copulatory organ that is inserted into the female (the embolus) is long and has coils corresponding to the number of coils in the female’s copulatory ducts. Each of the two copulatory ducts lead to a sperm storage organ, from which sperm leaves through a separate duct for fertilization [19]. If the somersault enables the male to push his embolus further into the female’s reproductive tract, higher paternity might be the result. In addition to releasing sperm deeper into the site of storage, the efficacy of placing a sperm plug that blocks rival males from acquiring paternity might also increase. In *Latrodectus,* the apical tip of the embolus can break off and remain lodged in the female’s genitalia. If the embolus tip blocks the entrance to the spermatheca, then subsequent mates are impeded from transferring sperm into the sperm storage organ, leading to high paternity for the first male to mate. If, however, insertion is shallower, the embolus tip may break off in the copulatory duct, where it will not function as an effective plug [29, 30].

Here, we test whether the somersault affects copulatory mechanics or the depth of insertion in *Latrodectus geometricus* by taking advantage of an alternative mating tactic of males that proceeds without the somersault. In both *L. geometricus* and *L. hasselti,* matings do not only occur between males and adult females, but also with immature females a few days before they moult to the adult stage (i.e. late subadult females; [31–33]) and this behaviour may be common in other *Latrodectus* [34]. In such “immature” matings, subadult females let the male approach and mount, then the male tears open the cuticle of the female at the genital area. This exposes the external female genitalia (epigyne) which, together with the copulatory ducts and spermatheca, are already well developed at this stage [31, 33]. In immature matings in *L. geometricus*, males do not somersault, they rarely somersault in *L. hasselti*, and in both species males always survive the mating [31]. Immature-mated *Latrodectus* females do not shed the ducts and spermathecae, in contrast to spiders that continue to moult after adulthood and shed the cuticular lining of the genitalia (e.g., liphistiids, mygalomorphs [15, 35]; but see [36] for an araneomorph). Therefore, they keep the sperm through the final moult and produce viable offspring of similar numbers as adult-mated females [31, 34]. Here, we investigated whether performing a somersault affects the coupling mechanism of the copulatory organs, allowing exploration of the interaction between behavior and genital mechanics. To this aim, we explored the interaction between male and female genitalia in adult (before and during somersaulting) and immature (without somersaulting) matings in the brown widow spider *L. geometricus*.

We hypothesized that the somersault affects (1) the genital coupling mechanism and (2) the depth of insertion of the male intromittent organ (i.e. embolus) into the spermatheca, which might affect sperm transfer and/or plug position. To test these hypotheses, we fixed spiders in copula, applied computed microtomography and reconstructed the male and female genitalia, the copulatory mechanism, embolus insertion depth and its position in the female genitalia. We explored the genital mechanism in matings with adult females, in the somersault position and also before the male flips over and compared the findings with the genital coupling in matings with subadult females where somersault behaviour does not occur. We predicted an advantage for somersaulting males that contributes to the selective benefits of this peculiar mating behaviour despite the entailed costs of cannibalism.

## Methods

### Experimental spiders

Adult females of *Latrodectus geometricus* C. L. Koch, 1841 (N = 10) were collected in Israel (Midrasha, Ramat Degev) and transported to the University of Greifswald, Germany, where they were kept in a climate chamber at 25 ± 1 °C under reversed 12:12 h light:dark conditions and 60% relative humidity. Egg sacs produced by the females were transferred to separate plastic containers (10 × 10 cm and 6 cm high). After emerging from the egg sac, spiderlings were kept together until their second moult, after which they were transferred into individual plastic containers in which they were reared. Spiderlings and adult males were fed fruit flies (*Drosophila hydei*) twice a week. Subadult females were fed with two *Lucilia sp.* flies, mature females with two *Protophormia sp.* flies twice a week. We monitored developing males and females and recorded the dates of their moults.

The females and males used for our mating experiments originated from different mothers. Adult females and adult males are readily recognized by the presence of developed external copulatory organs (epigyne) on the ventral side of the opisthosoma. Subadult females were used shortly (up to 6 days) before the final moult. These ‘late subadult females’ can be identified by the swelling and a colour change from grey to dark brown of the area that covers the developing external genitalia [31]. During this phase immature mating is possible when the male bites open the body wall covering the underlying genitalia [31–33].

### Morphology of copulatory organs

To illustrate the morphology of male and female copulatory organs when in resting position, pedipalps of three unmated adult males, and opisthosomata of two mated late-subadult female and two mated adult females were fixed in Duboscq-Brasil [37] and transferred to 80% ethanol.

### Mating trials—cryofixation

**Fig. 1:**
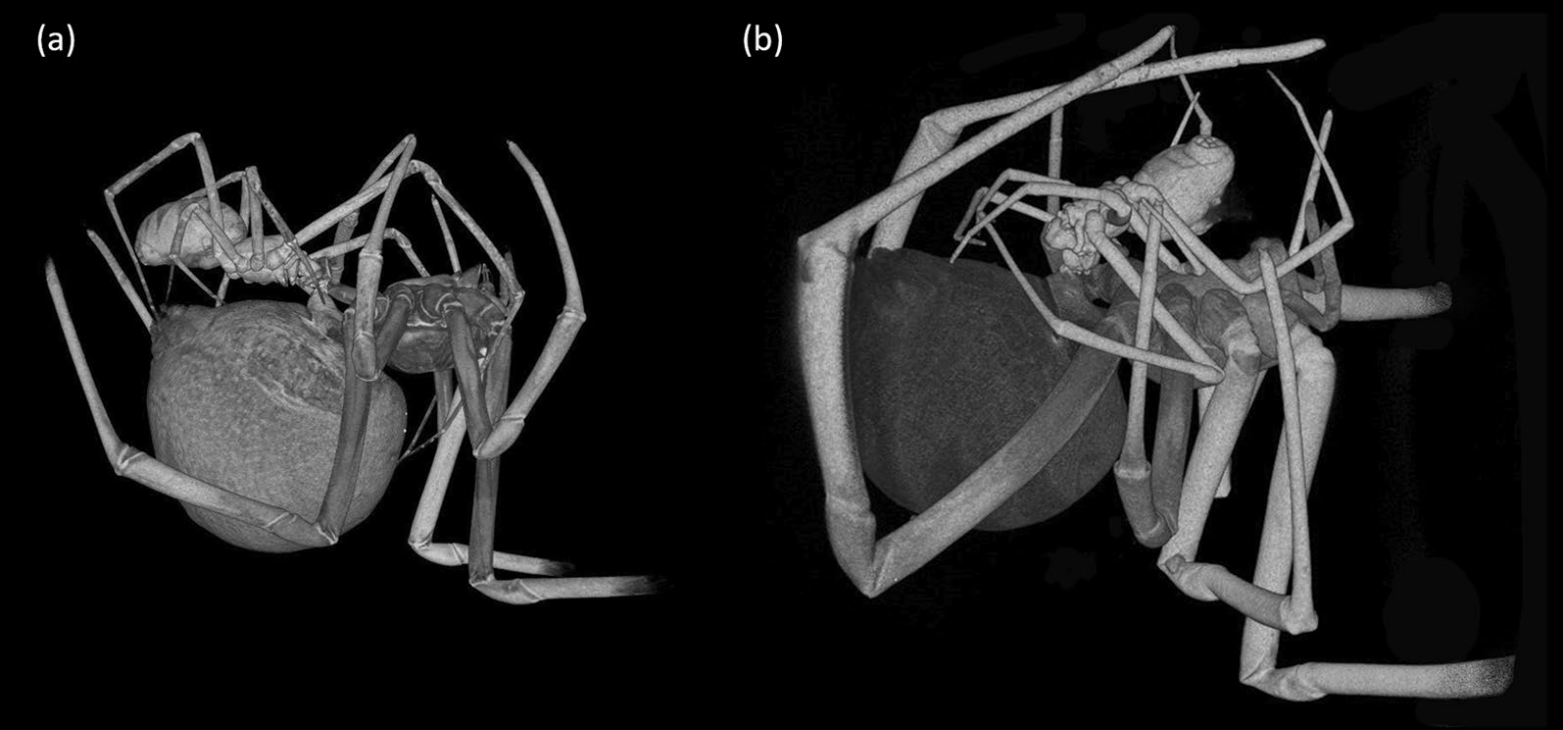
Mating position in immature **a** and adult mating after somersault **b**. The position of the male in adult mating prior to the somersault is the same as during the immature mating

To explore the interlocking mechanism of male and female copulatory organs, we staged matings. Two days prior to the trials, adult and late subadult females were transferred to clean experimental boxes (10 cm × 10 cm × 6 cm). The females were fed one day before the transfer and no food was provided while in the boxes. Since *L. geometricus* is nocturnal [38], the trials were conducted in the dark and males were introduced into the box at the beginning of the dark phase. The pairs were observed under a dissecting microscope with red light to track the progress of the copulation. At the beginning of an insertion the tip of male embolus is hooked into the entrance of female copulatory duct. Then the whole embolus is threaded through this long coiled duct in order to reach the spermatheca, which is a movement accompanied by shallow inflations of the membraneous parts of the male copulatory organ, the haematodocha. Only after the whole embolus is inserted, full inflations of the haematodocha were observed (L.S., personal observation), indicating sperm transfer [15]. We fixed the pair in copula only after full haematodochal inflations were observed to time the fixation when sperm transfer is already happening. We did so by pouring liquid nitrogen (− 196°C) over the pair in copula. The frozen couples were transferred to cold 80% ethanol and stored at − 40°C for three weeks to ensure stable fixation [39]. We successfully froze 17 couples during adult mating (11 in somersault position and 6 prior to the somersault) and 11 couples during immature mating (Fig. 1). Since a withdrawal of the embolus might have commenced during the process of freezing, we checked the frozen couples under a dissecting microscope to confirm that the embolus is fully inserted into the female copulatory duct. Such a full insertion was captured in 9 adult matings in somersault position (81.8%, N = 11), 6 prior to the somersault (100%, N = 6) and 8 during immature mating (72.7%, N = 11). Only the couples where a full insertion was achieved were further inspected.

### Computed microtomography

The samples were dehydrated in a graded ethanol series (three times in 80%, 90%, 96%, and 99% ethanol for 1 h each) and contrasted for 48 h using a 1% iodine solution (in pure ethanol). For scanning, the samples were critical point dried with a BAL-TEC CPD 030 and mounted on insect pins using super glue. Scans were performed in an Xradia Micro XCT-200 (Carl Zeiss Microscopy GmbH) using the macro lens (4x) for overviews of copulating pairs (scan parameters: 40 kV, 200 μA, exposure time 0.75—1.5, pixel size 3.98—5.56 µm) and the 10 × lens for close ups (scan parameters: 40 kV, 200 μA, exposure time 3.0—4.5 s/frame, pixel size 2.21–2.34 µm) on the interlocked copulatory organs. The male copulatory organs in resting position were scanned using a 20 × lens (scan parameters: 40 kV, 200 μA, exposure time 8–9 s/frame, pixel size 1.01—1.13 µm) and the female copulatory organs using a 10 × lens (scan parameters: 40 kV, 200 μA, exposure time 3.0—4.5 s/frame, pixel size 2.21–2.34 µm). Image stacks were created using XMReconstructor software (Carl Zeiss Microscopy GmbH). Data were visualized and processed using the 3D analysis software AMIRA 5.4.5 (Visualization Science Group, FEI).

**Fig. 2:**
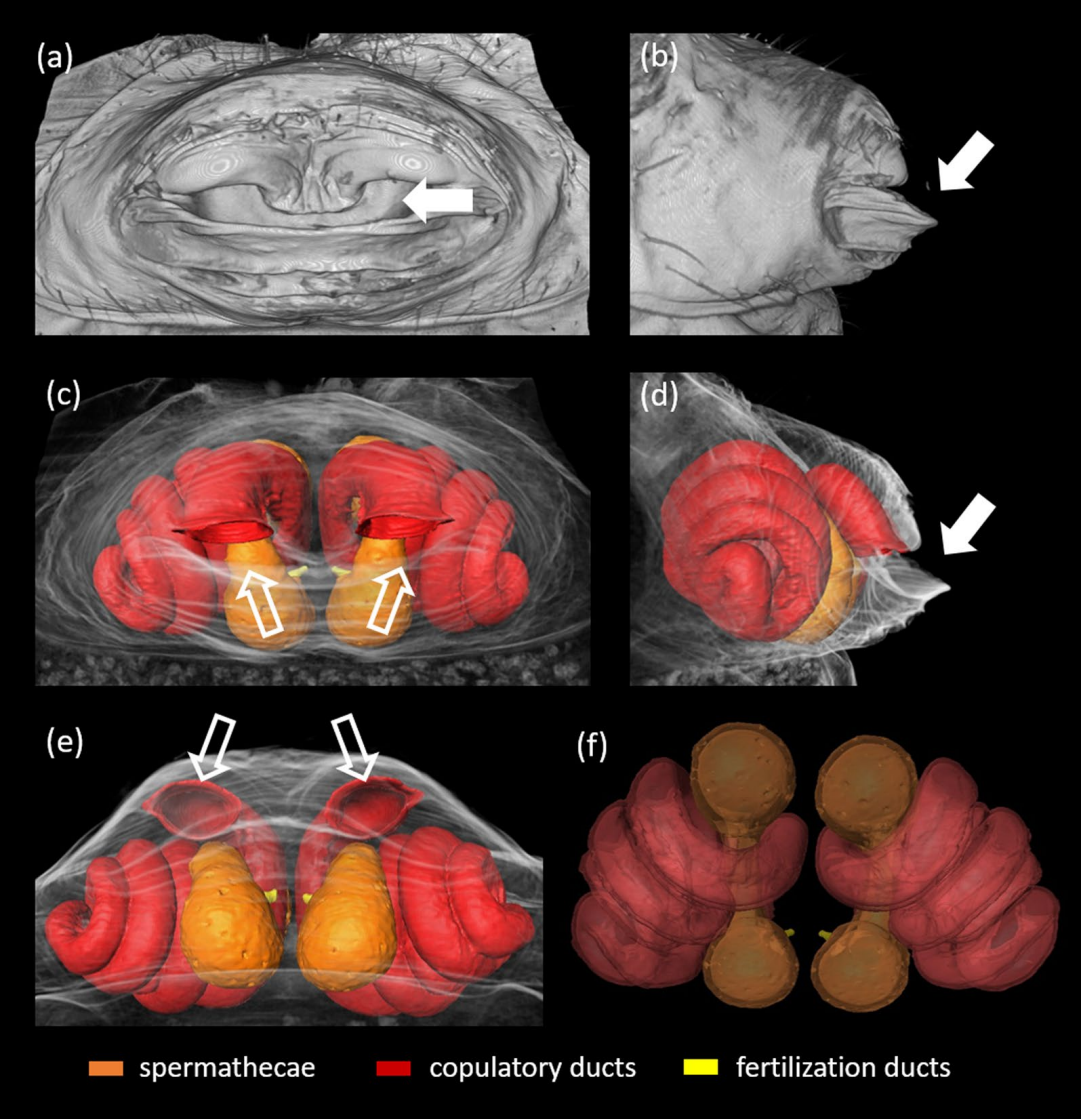
3D-reconstructed external (i.e. epigyne) and internal copulatory system (copulatory ducts, spermathecae and fertilization ducts) of a subadult *L. geometricus* female using MicroCT analysis. **a**. ventral and **b**. lateral view of the epigyne (volume reconstruction). **c**. ventral, **d**. lateral, **e**. axial and **f**. dorsal view of the internal copulatory system (surface reconstruction). The atrium (full arrows) hosts two copulatory openings (empty arrows) leading through copulatory ducts to spermatheacae

**Fig. 3:**
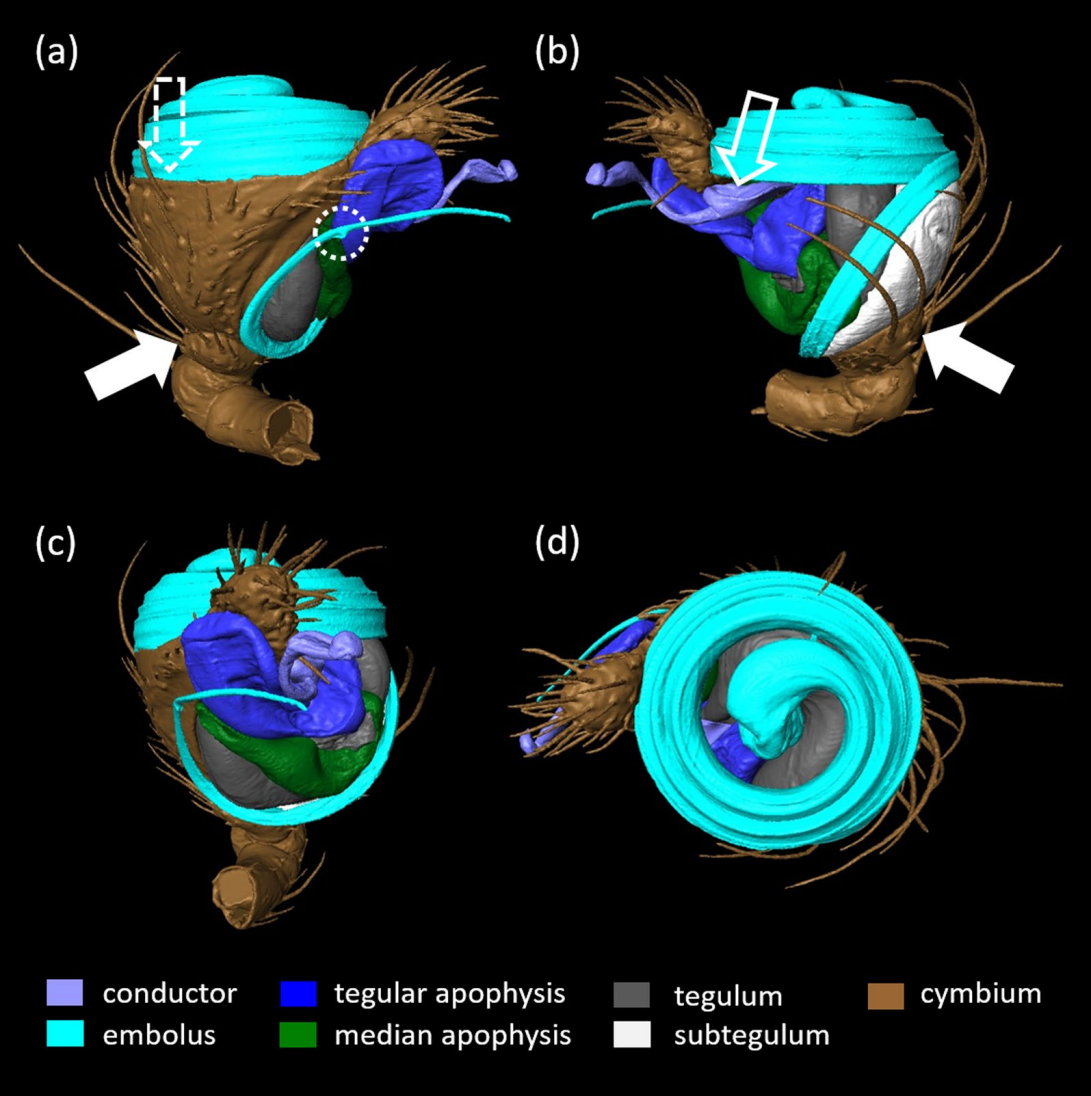
Surface reconstruction of the male copulatory organ of *L. geometricus*. **a**. prolateral view, **b**. retrolateral view, **c**. ventral view, **d**. apical view. Distal parts (embolus, conductor and apophyses) cover the basally located tegulum and subtegulum. The cup-like tibia (full arrow) carries the cymbium that is apically flattened, forming a furrow that hosts the basal loop of the embolus (dashed arrow). Close to its tip the embolus bears a thickening (dashed circle) where it typically breaks off during copulation. The basal part of the conductor forms a socket (empty arrow). Brown: cymbium and posterior podomers tibia, patella and part of femur

**Fig. 4:**
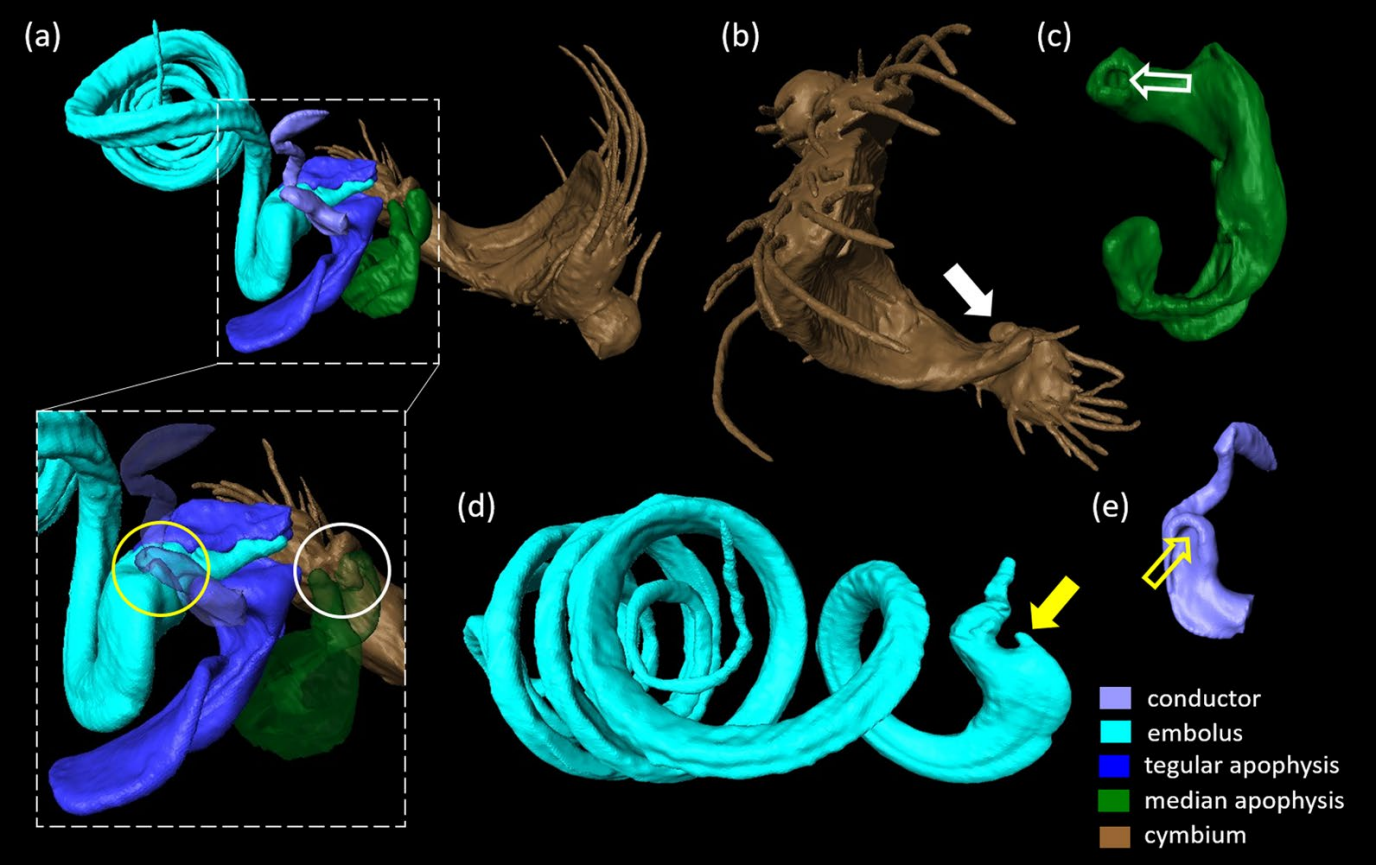
Bracing structures on the male pedipalp of *L. geometricus* and their position during copulation with adult females **a**. Relative position of select structures during mating. Inset shows the interlocking mechanisms in detail; transparency is used to show the underlying structures. **b** The cymbial furrow where the base of the embolus is rested when in resting position is visible during insertion (dashed arrow). The cymbium bears a cymbial hook (full white arrow in **b**) that interlocks with a hood (empty white arrow in **c**) on the median apophysis like a snap button (white circle). The embolus **d** bears a protrusion at its base (full yellow arrow) that hooks into the socket (empty yellow arrow) at the base of the conductor **e** (yellow circle in **a**)

**Fig. 5:**
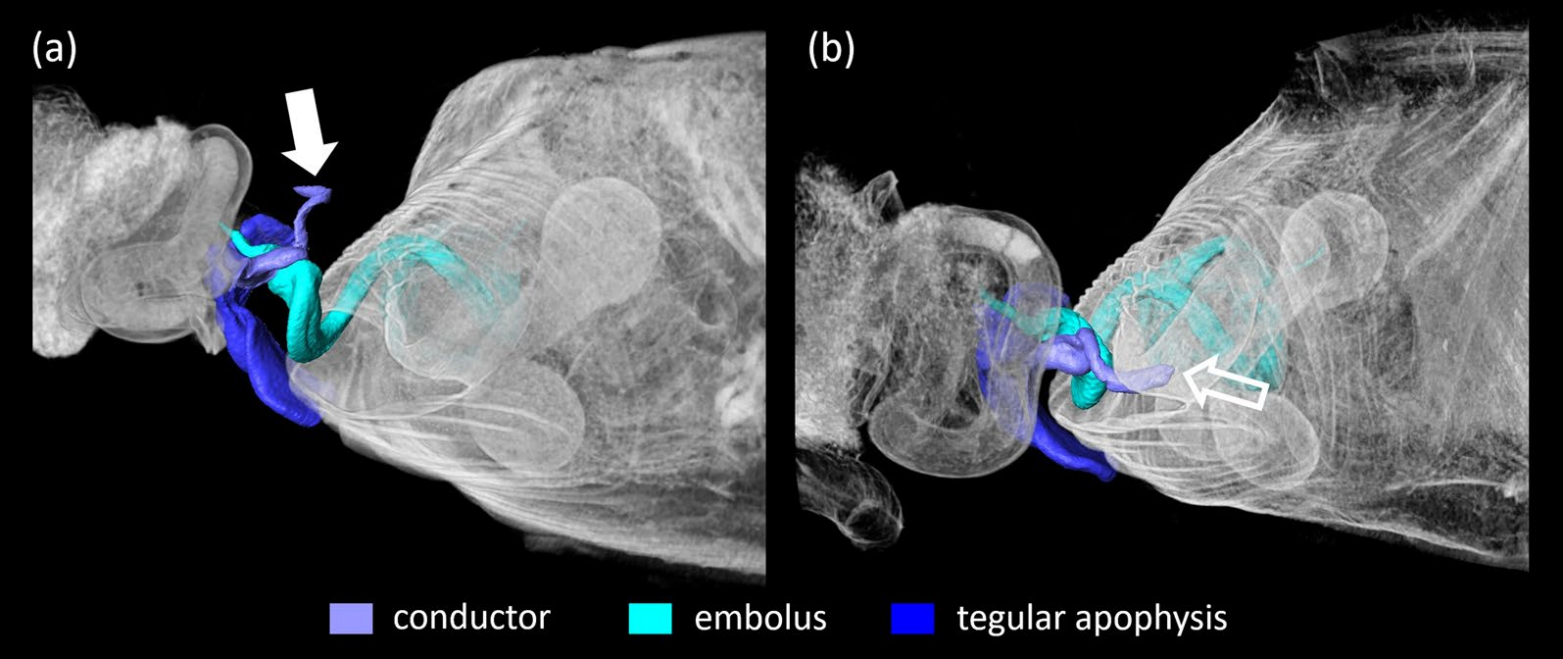
Different positions of the conductor during genital coupling in *L. geometricus* during matings with adult females. In most of the matings, we found the tip of the conductor to be not in contact with the epigyne (**a**, full arrow). In some cases, however, we found the conductor tip positioned inside the atrium (**b**, empty arrow)

**Fig. 6:**
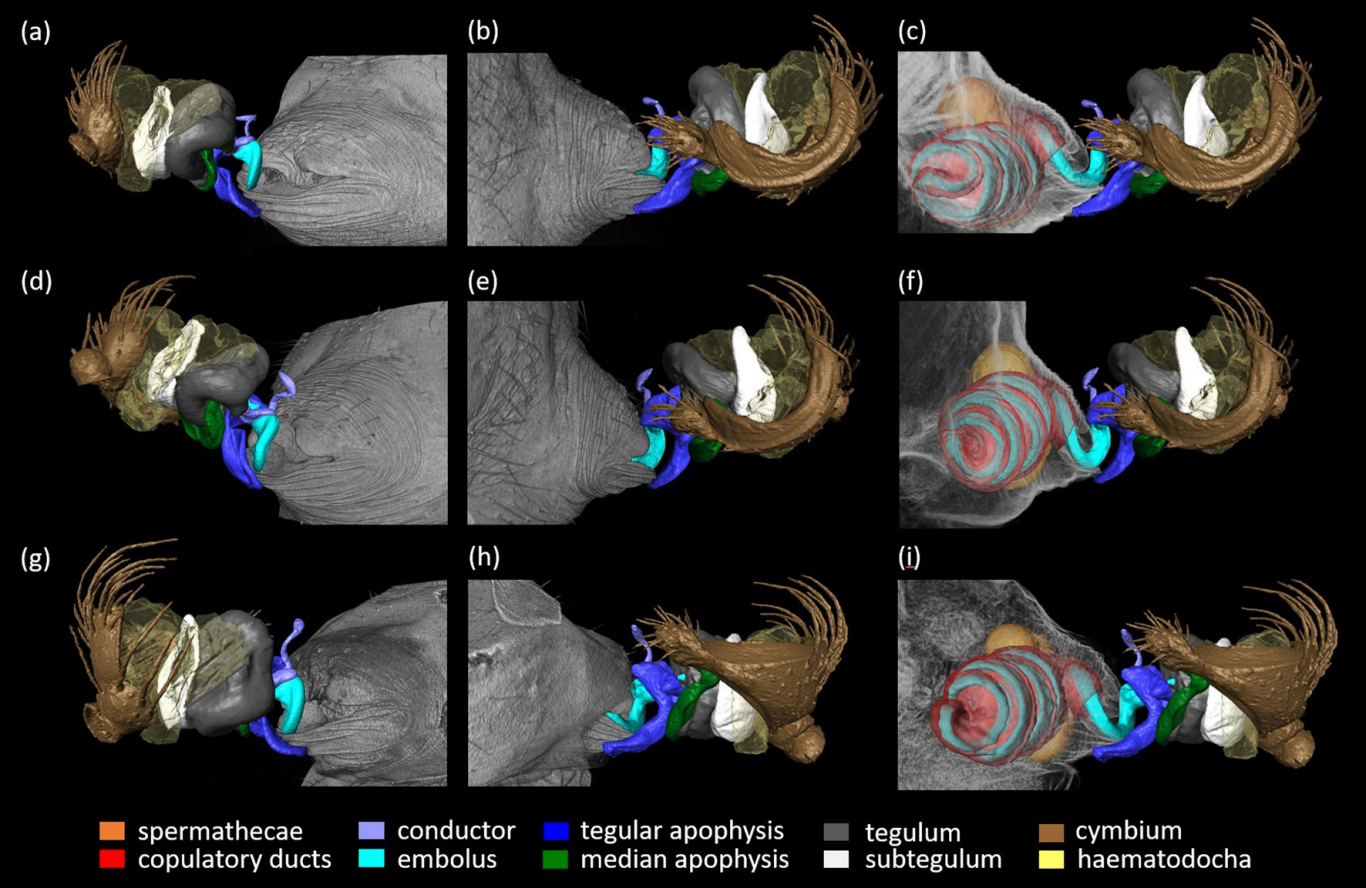
Coupling of the male and female copulatory organs in adult mating after copulatory somersault (**a**, **b**, **c**), in adult mating prior to somersault (**d**, **e**, **f**) and in immature mating (**g**, **h**, **i**) in *L. geometricus*. While the position of the cymbium and haematodocha slightly differed between cryofixed pairs, the apophyses and the embolus are locked in the same manner in all three positions/groups. The haematodocha is depicted as transparent because of its membranous nature

**Fig. 7:**
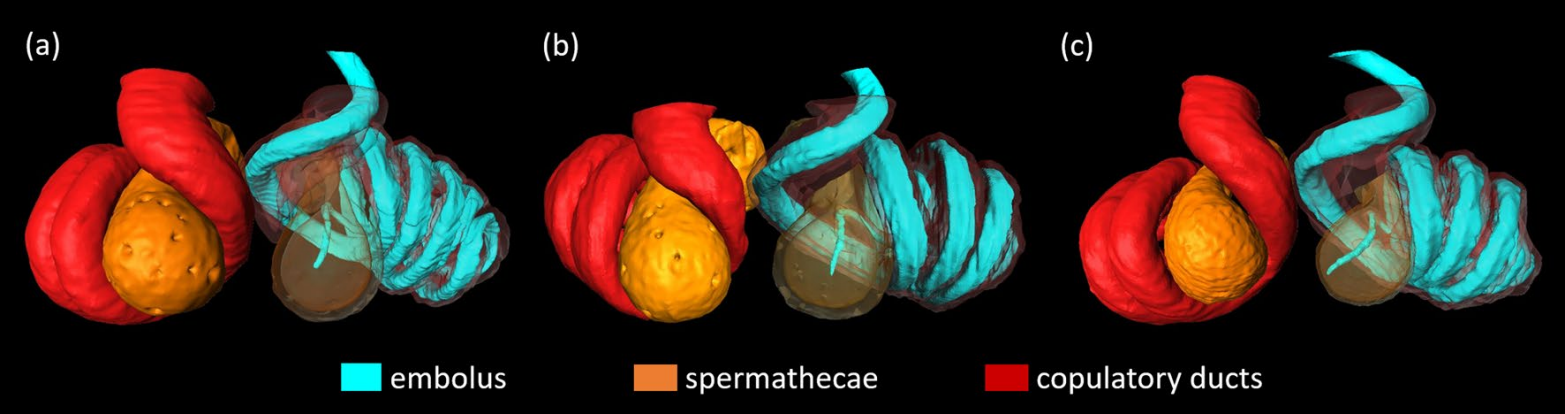
Insertion depth of the embolus in adult mating after the somersault **a**, prior to the somersault **b** and in immature mating **c**

First, we labelled and reconstructed male and female copulatory organ in resting position (Figs. 2 and 3). Then we labelled both male and female copulatory organs in the scans of frozen couples captured in full insertion. We described genital coupling during adult and immature mating focusing on the interaction of male and female copulatory organs (Figs. 4, 5, 6) and the position of the embolus within female copulatory tract (Fig. 7). We follow the terminology proposed by [40] for genitalia of Theridiidae.

## Results

### Female copulatory organ of *L. geometricus*

Late-subadult and adult females possess an external part of the female copulatory organ, i.e. epigyne, as well as internal copulatory system consisting of copulatory ducts, spermathecae and fertilization ducts (Fig. 2). In unmated late-subadult females, the epigyne is still covered by exocuticle, ready to be shed during the final moult to the adult stage (for more details see [33]). Males mating with late-subadult females bite through the cuticle exposing already formed epigyne and underlying copulatory structures [31]. In both late-subadult and adult females the epigyne is a protruding sclerotized plate traversed by an atrium, a cavity in which two copulatory openings are located (Fig. 2a–d—full arrow). The lower edge of the atrium forms a lip-like structure (Fig. 2b, d). Each copulatory opening (Fig. 2c, e—empty arrow) leads to a long, coiled copulatory duct that opens into one of the paired sperm storage organs, the spermathecae (Fig. 2c–f). Each spermatheca consists of anterior and posterior lobe connected by a narrow middle region (Fig. 2c–f). The copulatory duct opens into the anterior lobe, while the cuticle at the base of the posterior lobe forms the sclerotized beginning of the fertilization duct (Fig. 2c, e, f).

### Male copulatory organ of *L. geometricus*

A short, cup-shaped pedipalpal tibia (Fig. 3a, b—full arrow) carries an asymmetrical cymbium that ends in a distal protrusion (Figs. 3a-d, 4a, b). In the flattened middle region of the cymbium, a furrow occurs in which a portion of the embolus is rested (Figs. 3a—dashed arrow, c, 4a, b). The complex genital bulbus, consisting of several sclerites, emerges from the base of the cymbium to which it is connected by a membrane (i.e. haematodocha). The basal sclerites (subtegulum and tegulum), which are also connected by the haematodocha to each other, are hidden behind larger sclerites such as the median and tegular apophysis, the conductor and the intromittent, sperm transferring structure, the embolus (Fig. 3a–d). Within the bulbus a blind tube (i.e. spermophor) is situated. When the male reaches the adult stage, the sperm is transferred here via a sperm web from its site of production the testes in the opisthosoma. The sperm is taken up into the spermophor by the embolus which surrounds the distal part of the spermophor. The embolus is also used to transfer sperm into the female spermatheacae during mating [15]. The large and bilobate tegular apophysis is situated below the cymbial protrusion (Fig. 3a–c). The median apophysis is located below the tegular apophysis and only its median part is visible in the palp in resting position (Fig. 3b, c), as the tegular apophysis above obscures both of its ends, one forming a socket or a ‘hood’ (sensu [40]; Fig. 4a, c—empty white arrow). The conductor consists of a sclerotized base with a socket (Figs. 3b—empty white arrow; 4a, e—yellow empty arrow), and a less sclerotized, finger-like distal part (Figs. 3a–d; 4a, e; 5). The embolus is long and coiled (Fig. 3a, d) and wraps around the tegulum (Fig. 3a–d). Its basal loop rests on the cymbial furrow (Figs. 3a, c; 4b). Close to its tip, the embolus bears a thickening (Fig. 3a—dashed circle): this is the point at which it typically breaks off during copulation. These broken off tips are found in the female genitalia and form the mating plugs.

### Genital coupling in matings with adult females after somersault

Sclerites of the genital bulbus interact with each other to achieve structural stability of the intromission during mating (i.e. self-bracing mechanisms; [40, 41]; Fig. 4). There are several self-bracing mechanisms in *L. geometricus*: The median apophysis supports the base of the tegular apophysis and forms a bridge between the tegulum and the cymbium. The median apophysis is connected to the cymbium by a hood (Fig. 4b—empty white arrow) that embraces the cymbial hook (Fig. 4b—full white arrow) like a snap button (Fig. 4a—white circle). In all cryofixed pairs, the basal part of the conductor was found in a groove at the base of the embolus. Here, a socket-like structure on the conductor (Fig. 4e—empty yellow arrow) connects to a protrusion at the base of the embolus (Figs. 4a, d—full yellow arrow, e; 5; 6a, d, g).

The insertion mode of *L. geometricus* is ipsilateral meaning that the right embolus is inserted into right copulatory opening and the left embolus into left opening (Fig. 1). Studying the copulatory mechanism of adult individuals in somersault position, we found that the long, coiled embolus, is deeply inserted into the female copulatory duct (Fig. 6c) and that its tip reaches the posterior spermathecal lobe (Fig. 7a). The thicker base of the embolus is pressed outside against the region below the atrium. The tegular apophysis is pressed against the caudal lip-like edge of the epigyne and in concert, the base of the embolus and the tegular apophysis might pinch the epigyneal lip (Figs. 5, 6a–c). In some of cryofixed pairs (44.4%, N = 9), the apparently more flexible finger-like distal part of the conductor was located outside of the atrium—it protruded outwards and was not in contact with any female structure (Fig. 5a—full arrow). In three cases (33.3%, N = 9), however, the tip of the ‘finger’ was found inside the atrium (Fig. 5b—empty arrow) and in two cases (22.2%, N = 9) just outside of it. In all cases, a snap button connection exists between the conductor and embolus.

### Comparison of genital coupling in matings depending on mating type

The copulatory mechanism in matings with adult females after somersault is almost identical to that found in pairs cryofixed during immature mating and adult mating prior to the somersault. As mentioned above, in adult matings after the somersault, the position of the distal part of the conductor varied. In adult matings prior to somersault, it was located outside of the atrium in majority of cases (66.7%, N = 6). While its tip was never found inside the atrium, it was located just outside of it in two cases (33.3%, N = 6). In immature matings, the distal part of the conductor was positioned completely outside of the atrium in most cases (75%, N = 6), but in two cases, its tip was located inside the female atrium (25%, N = 8). Overall, the position of the distal part of conductor was not affected by female developmental stage or presence of the somersault.

The position of more proximal parts of the pedipalp (cymbium, subtegulum, haematodocha) varied slightly in position depending on the cryofixed pair (Fig. 6), regardless of whether the mating was with an adult or subadult female and regardless of whether a somersault was performed or not (Fig. 1, Fig. 6d–i). The membranous haematodocha, which is folded when the palp is in resting position, is inflated and deflated during sperm transfer as haemolymph is pumped into the genital bulbus (LS, personal observation), and we assume that these rhythmic pumping movements are responsible for this variation.

### Embolus position

Of all 26 cryofixed pairs, we were able to reliably track the insertion depth of the sperm transferring structure, the embolus, in six immature matings (out of N = 11, Fig. 7a), four adult matings in somersault position (N = 11, Fig. 7b) and three adult matings before somersault (N = 6, Fig. 7c). In all cases, the broken-off embolus tips reached all the way into the posterior lobe of the spermatheca with no difference between the groups (Fig. 7).

## Discussion

Males of the brown widow, *Latrodectus geometricus*, sacrifice themselves to the females as they rotate their opisthosoma onto female mouthparts following insertion of their intromittent organ (copulatory somersault). Although the position of male’s body changes dramatically during copulation due to the somersault, our study shows that the male copulatory organ remains firmly fixed into the female copulatory organ regardless of the relative positions of their bodies. In-copula freezing and microtomography revealed that the coupling mechanism of the copulatory organs is not externally or internally altered by the copulatory somersault. Correspondingly, the copulatory mechanism did not differ between adult and immature mating and adult mating before and after somersault. Our morphological data suggest that somersaulting did not evolve due to advantages from insertion depth and plugging efficiency. This conclusion is consistent with other work in which male *L. geometricus* that mate subadults (with no somersaulting) achieve higher plugging success than males that somersault while mating with adult females [28, 31, 33], reinforcing the inference that the somersault is not required for deep and effective insertions.

The mating position in spiders is generally family-specific [13, 14] but can vary drastically within theridiids. Most theridiid spiders assume the typical web spider mating position—an antiparallel mating posture, with the venter of both sexes facing upwards [42]. However, in *Latrodectus* and other theridiids with strong sexual size dimorphism, the much smaller males approach the females from behind and insertion of the pedipalps takes place venter to venter, with both sexes facing in the same direction [42, 43]. In *Latrodectus* males that somersault, the males flip their body over while their pedipalps remain arrested in copulation [20]. Our study shows that flipping over does not change the connection between male and adult female copulatory organs. Similarly, in matings with subadult females with no somersaulting, the copulatory mechanism is the same as in adult matings.

The insertion pattern of *L. geometricus* is ipsilateral as in most ‘Entelegynae’ spiders [44], but different from other theridiids that show contralateral insertions (e.g. genus *Theridion*; see [45]). The tibia of the male pedipalp in the family Theridiidae is cup-like, with a constricted base (see [40]). This conserved tibial morphology might allow a certain flexibility of the angle at which the male pedipalp is connected to the female during genital coupling, thus enabling the acquisition of particular mating behaviors—such as the somersault.

The copulatory mechanism in *Latrodectus* itself entails several interlocking and bracing aspects. Such interlocking devices are abundant in spiders as are bracing mechanisms between male and female structures (e.g. [4, 46, 47]). In *L. geometricus*, the interaction of the cymbial hook with the hood of the median apophysis seems to grant structural stability for the inserted palp as it locks the bulb in the pedipalp and allows to control expansion of the haematodocha during sperm transfer [40]. The cymbial hook is a theridiid synapomorphy; it holds the same function in the genus *Theridion* [45] and probably in all Theridiidae [40]. Additionally, the “snap button” connection between the conductor base and the embolus base in *L. geometricus* further stabilizes the coupling position. Our study also shows, that the tegular apophysis does not support the embolus as in many other theridiid spiders [40] but that these two sclerites act together as pincers gripping the lower lip-like edge of the epigyne, thereby anchoring the palp to the female external copulatory organ. We conclude that once the male genital structures are interlocked, inserted and anchored on the female epigyne, the males can change their body position without altering the coupling mechanism. This ability might be an intrinsic feature of theridiids, as in some *Crustulina* and *Steatoda* species, the female rotates 180º when genital contact is achieved, leading to atypical mating positions [42]. The fact that the genital coupling is sustained in these cases, and during the somersault of *L. geometricus*, suggests that theridiid male pedipalps can resist abrupt flipping or twisting of the male´s body without altering the position of the coupled genitalia.

The tight interlocking mechanism also extends to the sperm transferring structure in *L. geometricus*. Insertion depth of the embolus does not differ before and after the somersault in adult matings. Surprisingly, it also does not differ from that in matings with subadult females, although the cuticle of the genital region is likely still soft and the cuticle of the sperm storage organ is not yet fully developed (1/3 of the thickness of that of adult females; [33]). Despite the softness of the tissue involved, we did not detect difficulties in genital coupling and differences in insertion depth. This suggests that the interlocking mechanism between male sclerites guarantees successful coupling even if the female counterparts are not as rigid as in adult females.

The embolus of widow spiders is a long, coiled, heavily sclerotised structure that is threaded through the female copulatory duct during copulation. Close to the tip of the embolus, a small hump marks the break-off point during the copulation [30, 48]. If a male succeeds in inserting the embolus, the hump is typically situated at the end of the copulatory duct with the tip of the embolus extending into the lumen of the spermatheca. When the embolus breaks off, the hump likely anchors the embolus tip in this position [30, 48]. Changes in the mating position such as a somersault could theoretically change the position of the embolus. If the embolus ends up deeper in the female copulatory tract in a way that the male has a higher chance of sperm transfer and a higher chance that the embolus tip blocks the entrance to the spermatheca, somersaulting would be selectively favoured. However, in *L. geometricus* the embolus did not reach deeper into the female spermatheca in couples fixed in somersault position (i.e. in adult females), which makes the behavior a significant puzzle in this species. First, males that somersault may trigger sexual cannibalism already during the first insertion. The female typically starts to masticate the opisthosoma at this point, and some males are killed, leaving the opposite spermatheca unused and unplugged, which could be a disadvantage for such males. This may not be a cost, however, as most males survive to mount the female again and inseminate the other spermatheca [21, see 49 for a similar result in *L. hasselti*]. However, unlike *L. hasselti* [26] the somersault in *L. geometricus* does not reduce the probability that the female will mate with another male [28]. Additionally, in *L. geometricus* the somersault shortens [28] rather than prolongs the copulation as found in *L. hasselti* [26]. Therefore, the previous studies focusing on behavioural observations also did not clarify why male engage in this behaviour in *L. geometricus*. Our morphological data suggest that somersaulting did not evolve due to advantages from insertion depth and plugging efficiency.

## Conclusions

We conclude that somersaulting must provide other advantages, such as differential sperm storage and utilisation, which are likely connected to cannibalism. Sperm competition experiments using rival males in different settings (e.g. using a combination of natural scenarios with females that experienced one or two insertions in immature matings as well as later matings as adults) may elucidate if there is differential reproductive success depending on whether immature mating occurred and whether or not a somersault was performed. Overall, our results suggest that the biomechanics of copulation are largely robust to variations in male copulatory behavior, even when that behaviour involves an extreme change in mating posture.

## Data Availability

The datasets (scans) supporting the conclusions of this article are available in the Zenodo repository https://doi.org/10.5281/zenodo.14772376.
